# Fully angularly resolved 3D microrheology with optical tweezers

**DOI:** 10.1007/s00397-024-01435-1

**Published:** 2024-02-08

**Authors:** Andrew B. Matheson, Tania Mendonca, Matthew G. Smith, Ben Sutcliffe, Andrea Jannina Fernandez, Lynn Paterson, Paul A. Dalgarno, Amanda J. Wright, Manlio Tassieri

**Affiliations:** 1 School of Engineering and Physical Sciences, Institute of Biological Chemistry, Biophysics and Bioengineering, Heriot Watt University, Edinburgh, UK; 2 Optics and Photonics Research Group, Faculty of Engineering, University of Nottingham, Nottingham, UK; 3 Division of Biomedical Engineering, James Watt School of Engineering, University of Glasgow, Glasgow, UK

**Keywords:** Microrheology, Computer modeling, Simulation, Viscosity

## Abstract

Microrheology with optical tweezers (MOT) is an all-optical technique that allows the user to investigate a materials’ viscoelastic properties at microscopic scales, and is particularly useful for those materials that feature complex microstructures, such as biological samples. MOT is increasingly being employed alongside 3D imaging systems and particle tracking methods to generate maps showing not only how properties may vary between different points in a sample but also how at a single point the viscoelastic properties may vary with direction. However, due to the diffraction limited shape of focussed beams, optical traps are inherently anisotropic in 3D. This can result in a significant overestimation of the fluids’ viscosity in certain directions. As such, the rheological properties can only be accurately probed along directions parallel or perpendicular to the axis of trap beam propagation. In this work, a new analytical method is demonstrated to overcome this potential artefact. This is achieved by performing principal component analysis on 3D MOT data to characterise the trap, and then identify the frequency range over which trap anisotropy influences the data. This approach is initially applied to simulated data for a Newtonian fluid where the trap anisotropy induced maximum error in viscosity is reduced from ~ 150% to less than 6%. The effectiveness of the method is corroborated by experimental MOT measurements performed with water and gelatine solutions, thus confirming that the microrheology of a fluid can be extracted reliably across a wide frequency range and in any arbitrary direction. This work opens the door to fully spatially *and* angularly resolved 3D mapping of the rheological properties of soft materials over a broad frequency range.

## Introduction

Microrheology techniques are a highly effective and versatile family of tools that allow experimentalists to analyse the free or driven motion of probe particles suspended in a complex media of interest. From this motion, the viscoelastic properties of materials at micron and sub-micron length scales can be extracted. These techniques are typically classified as either ‘active’ or ‘passive’ microrheology depending on whether the motion of the probe particle is induced by an external force field or by the thermal fluctuation of the molecules of the suspending media, respectively. In the case of microrheology with optical tweezers (MOT), sometimes referred to as a hybrid approach, a tracer bead or other probe particle is optically trapped by a highly focussed laser beam. Although held by the trap(Ashkin, et al. 1986), the bead still undergoes Brownian motion within a finite volume defined by the trap, and its trajectory can be traced and analysed to determine the viscoelastic properties of the suspending media (Furst 2005; Kumar, et al. 2021; Meyer, et al. 2006; Nemet and Cronin-Golomb 2003; Pesce, et al. 2005). This has proved to be a highly effective technique, especially for the analysis of biological samples (Ashworth, et al. 2020; Guadayol, et al. 2021; Mao, et al. 2022; Rizzi and Tassieri 2018; Watts, et al. 2013; Weihs, et al. 2006; Xing, et al. 2018).

**Fig. 1:**
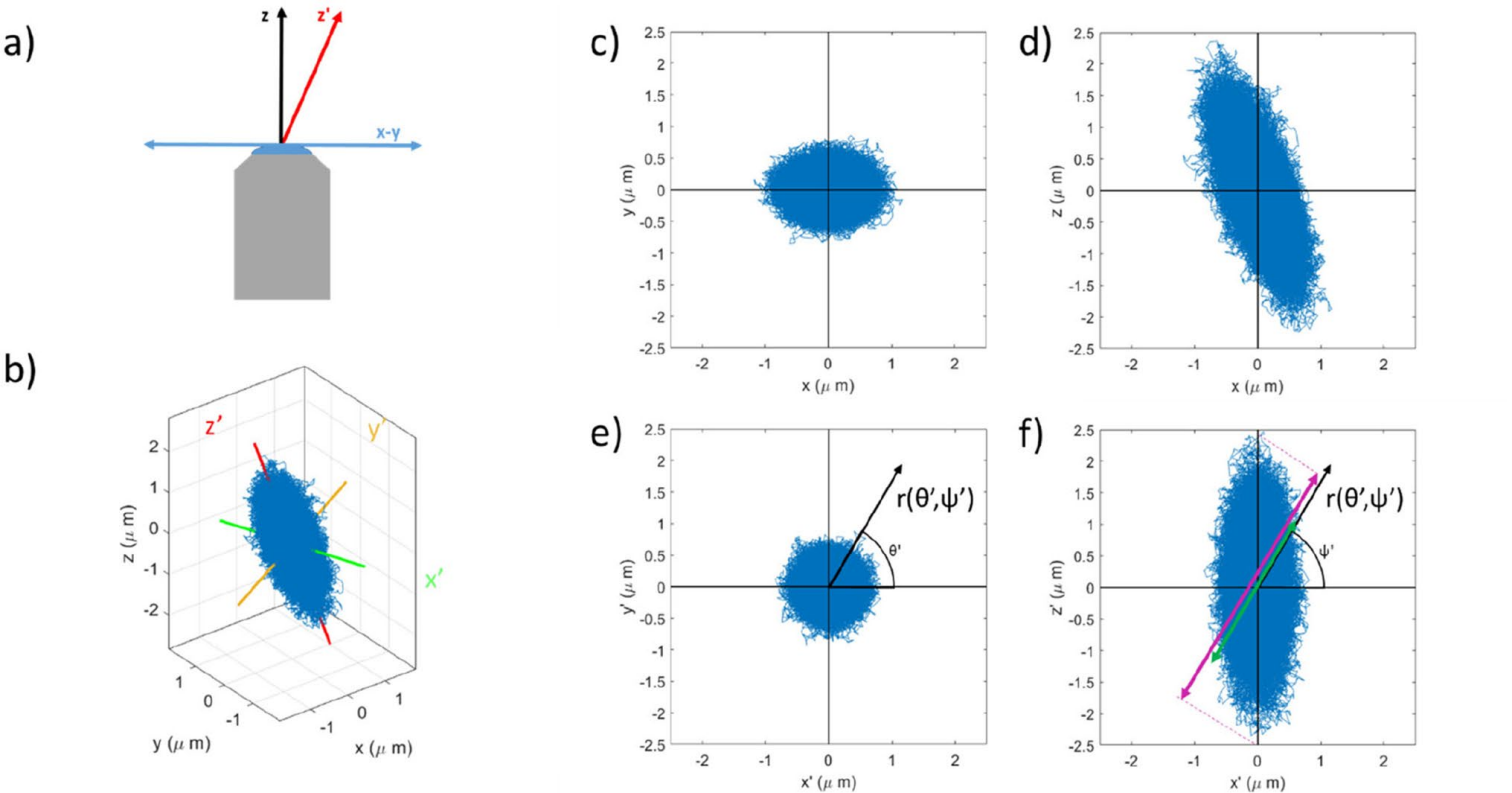
**a** Schematic representation of a misalignment between the optical axis of the imaging system (*z*, shown in black), and the axis of trapping beam propagation (*z*′, shown in red), perpendicular to *z* is the plane being imaged (*x*–*y*, shown in blue). **b** Simulated 3D trajectory of a bead with ${\kappa }_{x'} = {\kappa }_{y'}$ = 1 × 10^−7^ N m^−1^, ${\kappa }_{z'}$ = 1 × 10^−8^ N m^−1^ and *z*′ at 20° to *z*. The *x*′, *y*′, and *z*′ axes are shown in green, yellow, and red, respectively. **c**–**d** The projections of the bead trajectory on the *x*–*y* and *x*–*z* planes, respectively. The projections of the same trajectory on the *x*′-*y*′ and *x*′-*z*′ planes are shown in **e** and **f**, respectively, together with the vector $r({\theta'},{\psi'})$ used for the microrheology analysis. The purple arrow in **f** gives an approximate measure of the spread of coordinates along $r({\theta' },{\psi'})$ due to the trap geometry, whereas the green arrow shows the maximal spread expected for a symmetric trap

Typically particle tracking, and therefore MOT, is carried out using the 2D projection of the trajectory across the imaging plane of the microscope (often referred as the *x*–*y* plane, see Fig. 1a) (Ciccone, et al. 2020; Tassieri 2019). However, several different tracking approaches have been developed for performing microrheology in 3D (Liang, et al. 2020; Matheson, et al. 2021b; Rohrbach and Stelzer 2002). This theoretically offers the possibility of probing the viscoelastic properties of the sample in all directions simultaneously. This is particularly appealing for biological samples where viscoelastic properties will vary spatially in all three dimensions, and could be highly anisotropic at a given point (Hasnain and Donald 2006; Mendonca, et al. 2023). Furthermore, it is well established that in the proximity to an interface, the viscosity as obtained from microrheology may vary significantly with angle (Leach, et al. 2009; Schäffer, et al. 2007), providing an additional possible application for full angular resolution.

Moreover, in the field of microrheology, it is desirable to obtain measurements over the widest range of experimentally accessible frequencies. The latter has an upper limit dictated by the acquisition rate of the imaging system and the resolution of the particle tracking system. For MOT, the minimum frequency that can be accessed experimentally is closely linked to the so called ‘corner frequency’ (${f}_{c}$) of the system:

1 $${f}_{c}=\frac{\kappa }{6\pi \eta a}$$

where $\kappa$ is the trap stiffness, $\eta$ is the fluid viscosity, and $a$ is the probe radius. Therefore, for any given combination of bead probe and material, the lowest accessible frequency can only be extended by reducing $\kappa$ (typically by lowering the power of the laser). However, there is a delicate balance to be struck, because if $\kappa$ is set too low, it will not be possible to optically trap the bead and the bead will diffuse out of the field of view while the measurement is being made. Tracking in 3D offers a route to extend the low frequency range of MOT measurements. Due to diffraction and the fundamental physics underpinning optical trapping, under normal operating conditions (i.e., when the laser intensity profile is a Gaussian beam), the trap strength is weakest along the axis of laser propagation and strongest in the plane perpendicular to the direction of laser propagation. (Ashkin, et al. 1986). Though the axis of laser beam propagation *z*′ (see Fig. 1a) will not necessarily be perfectly coincident with the axis perpendicular to the imaging plane of the system, *z*, in all but the most extreme cases of misalignment, *z*′ will be much closer to *z* than to *x*–*y*. Therefore, by switching from tracking in 2D (*x*–*y* plane) to 3D (*x*, *y*, and *z*) one can gain access to lower frequencies (as ${\kappa }_{z}<{\kappa }_{x, },{\kappa }_{y}$) without the need to reduce the trap strength of the system and thus risk losing the bead.

In recent work from this group, it has been shown that using the standard 2D analytical tools on a trap with a degree of anisotropy of 30% or higher can result in a substantial underestimation of the trap strength, and an overestimation of the fluid viscosity in certain directions (Matheson et al. 2021a). Crucially, as a consequence of diffraction and the focal spot being stretched along the axis of laser beam propagation, the difference in trap strength between ${\kappa }_{z'}$ versus ${\kappa }_{x'}$ and ${\kappa }_{y'}$ has been observed to exceed this level of anisotropy (Matheson et al. 2021a, Matheson, et al. 2021b; Mendonca, et al. 2023). This is without even considering other aberrations in the optical system may also introduce additional sources of trap anisotropy (Bowman, et al. 2010; Dutra, et al. 2007, 2012; López-Quesada, et al. 2009; Roichman, et al. 2006). The simplest solution to this problem is to identify the directions for which the variance in the bead displacement, and hence, $\kappa$ is either at its maximum or minimum, as along these directions the estimate of trap strength should be accurate and discrepancies in the microrheology measurement minimised (Matheson et al. 2021a). In practice, this means identifying and then performing the analysis only along the direction of *z*′ and the orthogonal plane *x*′-*y*′, which for a well aligned trap should be equivalent to *z* and *x*–*y*, respectively. However, this means that the bead trajectory along all other directions is then disregarded, prohibiting properties such as viscosity, and storage and loss moduli from being fully angularly resolved. To the best of our knowledge, an analytical approach that allows microrheological properties to be accurately probed in *any* direction in 3D using MOT is still missing from the literature. In this work, we present such a method for evaluating the rheological properties of fluids from MOT measurements fully in 3D first testing it on simulated data, and then confirming its effectiveness on water and gelatine samples. This will allow for more rapid identification of *z*′ than in previous work (Matheson et al. 2021a), and help correct for artefacts due to misalignment. More significantly, whereas in previous work, we described how anisotropy may cause artefacts in MOT results (Matheson et al. 2021a), and how to find the angles where these artefacts will not be present; here, we have developed a method to avoid these artefacts completely at any angle. This will allow for the researchers to accurately measure the viscoelastic properties of a material in any arbitrary direction, rather than being constrained to just *z*′ and the *x*′-*y*′ plane.

## Experimental methods

### Microrheology with optical tweezers

The optical trap was generated using a 1064-nm DPSS laser (Opus, Laser Quantum) with a maximum output of 5W on an inverted microscope set up (Olympus IX-73) with a high NA objective lens (LUMFLN60XW 60 × 1.1 NA 1.5 mm WD; Olympus). For tracking in 3D, a multiplane imaging system utilising a pair of gratings to produce nine images spatially separated on the image sensor each with a different focal plane was employed, the details of which are described elsewhere (Matheson et al. 2021a, Matheson, et al. 2021b; Mendonca, et al. 2023). In brief, a 4f image relay system consisting of two 300 mm lenses was set up in the detection path between the camera (Hamamatsu ORCA Flash 4.0 V2) and the microscope body, and the multiplane grating pair was placed in the telecentric position. The relay and grating combination used here gives a plane separation of Δ*z* = 0.79 μm with the nine images spanning 7.11 μm. The constrained Brownian motion of the trapped 6 μm diameter microsphere probe was recorded using MicroManager (version 1.4) and analysis carried out in Matlab (R2021b, Mathworks) to extract trajectories. A full description of the particle detection system can be found in Matheson, et al. (2021b), but in brief, centre of mass is used to calculate *x* and *y* coordinates, while a combination of centre of mass and image sharpness are used to calculate the *z* position. The resolution of these techniques are approximately 15 nm in *x* and *y*, and 30 nm in *z*. Temperature control was provided via the laboratory air conditioning system, with temperature monitored using a digital thermometer to ensure thermal stability.

All the experiments used 6 μm diameter beads (Polybead® Microspheres 6.00 μm; PolySciences) as the trapped probe particle. For the water experiments, beads were dispersed in an 8 u-well glass bottom coverslip (ibidi) at a final dilution of 1:500,000 from a stock concentration of 2.1 × 10^8^ particles/ml. For gelatin experiments, gelatin powder (Sigma-Aldrich, 48,723-500G-F, Lot# BCBW0732) was dissolved in warm distilled water and beads were dispersed at a final dilution of 1:25,000 in the solution. This was then placed in a water bath at 70 °C for 15 min while being sonically agitated. The gelatin and bead solution (300 μl) was then added to each well of a 8 u-well glass bottom coverslip (ibidi) and left to set for 24 h at 4 °C. In both case, beads were trapped > 40 μm from the coverslip.

### Computational simulation

Monte Carlo simulations were carried out using Matlab (R2021b, Mathworks) code based on the work presented by Volpe et al. (Volpe and Volpe 2013) and following the approach described by Matheson et al. (Matheson et al. 2021a) but extended into 3D. To briefly summarise, thermal forces are simulated to conform to a Boltzmann distribution, acting along the *x*′, *y*′, and *z*′ axes. There are corresponding restorative forces which then accelerate the bead back towards the centre of the trap. For the simulations shown in Fig. 1, the parameters used were ${\kappa }_{x'} = {\kappa }_{y'} =$ 1 × 10^−7^ N m^−1^, ${\kappa }_{z'}$ = 1 × 10^−8^ N m^−1^ and *z*′ was at 20° to *z*, with a bead radius of 1 μm and a solvent simulated to have the same viscosity as water at 20 °C. These parameters were chosen to be of the same order of magnitude as those typically present in microrheology measurements, such as the experimental data presented in this paper.

### Bulk rheology

Bulk rheology measurements of water and gelatine were performed by using a single head stress-controlled rheometer (Anton Paar MCR 302) equipped with a cone-plate measuring system (CP60-1-SN42255). The temperature was controlled by means of a Peltier system connected to a water bath. The viscosity of the fluid was measured by performing a flow curve test at shear rates varying from 50 to 500 s^−1^. For both the samples, seven replicates were recorded and the mean average taken.

## Theoretical background

If one begins by considering a bead trajectory of the form shown in Fig. 1b, the scatter plot is clearly elongated along an axis *z*′, and narrowest in the plane defined by *x*′-*y*′. It can therefore be described as anisotropic. The scatter plot resembles an ellipsoid with *x*′, *y*′, and *z*′ as the semi-axes. For this trap, *z*′ (the axis of trap propagation) is not colinear with *z* (the axis of the imaging system), so as well as being anisotropic, the trap is misaligned. This results in an oval cross-section in *x*–*y* (Fig. 1c) and a clear tilt in *x*–*z* (Fig. 1d), despite being circular in *x*′-*y*′ (Fig. 1e) and symmetric in *x*′-*z*′ (Fig. 1f). Trap anisotropy is an unavoidable consequence of the Abbe diffraction limit, and will be present in all trap beams. Trap misalignment can be avoided, but is not always easy to spot when detection is only carried out in 2D. However, provided the trap is well aligned, the resulting scatter plot is isotropic if one is only considering the projection of coordinates onto a 2D plane (as in Fig. 1e), as is the norm for most MOT measurements. When looking at the full 3D scatter-plot, this is not the case and an anisotropy in the trajectory is unavoidable. The following describes the consequences of this, and how to mitigate any artefacts it causes.

### Estimating the trap stiffness in 3D

For optical tweezers, the trap stiffness is calculated by appealing to the principle of equipartition of energy:

2 $${\kappa }_{Eq}\left({\theta'},{\psi'}\right)=\frac{{k}_{B}T}{<{r}^{2}({\theta'},\psi')>}$$

where ${k}_{B}$ is the Boltzmann constant, $T$ is the absolute temperature, and $\langle {r}^{2}({\theta'},\psi')\rangle$ is the variance of the particle trajectory $r(t,{\theta'},{\psi'})$. Here, the subscript ‘Eq’ is used to differentiate it from the other means of calculating $\kappa$ outlined later. It has been shown that Eq. 2 gives spurious results when analysing $\langle {r}^{2}({\theta'},\psi')\rangle$ if it is neither in the *x*′-*y*′ plane or aligned with *z*′ (Matheson et al. 2021a). This is because in these cases, motion along the direction being analysed (defined by the unit vector $\widehat{r}({\theta'},{\psi'}$, shown in Fig. 1 e and f) becomes correlated with motion in the directions orthogonal to $\widehat{r}({\theta'},{\psi'})$. This implies that the value of $\langle {r}^{2}({\theta'},\psi')\rangle$ is no longer defined purely by the trap stiffness $\kappa ({\theta'},\psi')$ aligned in the $\widehat{r}({\theta'},{\psi'})$ direction but also by the trap stiffness along the orthogonal directions. This is represented graphically by the green and purple arrows in Fig. 1f. It follows that a different approach must be devised for calculating $\kappa ({\theta'},{\psi'})$, as elucidated hereafter.

The restoring force acting on a bead along any direction defined by *θ*′ and *ψ*′ can be described as:

3 $$F\left(t,{\theta'},{\psi'}\right)= -\kappa \left({\theta'},{\psi'}\right)r\left(t,{\theta'},{\psi'}\right)$$

By considering the force along the $\widehat{r}({\theta'},{\psi'})$ direction to be the vector sum of the forces acting along the *x*′, *y*′, and *z*′ axes, the trap strength along $\widehat{r}({\theta'},{\psi'})$ direction is defined as follows:

4 $${\kappa }_{{\text{Force}}}\left({\theta'},{\psi'}\right)={\left({\left({\kappa }_{x'}{\text{cos}}\left({\theta'}\right){\text{cos}}\left({\psi'}\right)\right)}^{2}+{(\kappa }_{y'}{\text{sin}}\left({\theta'}\right){{\text{cos}}\left({\psi'}\right))}^{2}+{(\kappa }_{z'}{{\text{sin}}\left({\psi'}\right))}^{2}\right)}^\frac{1}{2}$$

Hence, the need to find the direction of propagation of the laser beam, which here is defined as *z*′, so that *x*′, *y*′, and *z*′ can be properly identified. In order to identify *z*′, we perform a principal component analysis (PCA) of the particle trajectory, which here has been implemented by using the built in ‘pca’ function in Matlab. This routine returns the principal component coefficients of the data. The first principal component corresponds to *z*′, i.e., the axis with the largest possible variance in the particle trajectory. The other two principal components are orthogonal to the first principal component and represent *x*′ and *y*′ in our 3D distribution.

Once *x*′, *y*′, and *z*′ are identified, it is a trivial step to calculate the trap strength acting along these directions by using Eq. (2), and then to calculate the trap strength in any arbitrary direction via Eq. (4). In order to obtain ${\kappa }_{Eq}({\theta'},{\psi'})$ at the necessary angles, the trajectory can be scanned in 5° intervals of the angles *θ*′ and *ψ*′ around the *z*′ and *y*′ axes, respectively. In practice, this is achieved by iteratively performing linear coordinate transformations of the particle coordinates and then sampling the transformed data along the *x*′ axis, as shown in Fig. 1 e and f.

**Fig. 2:**
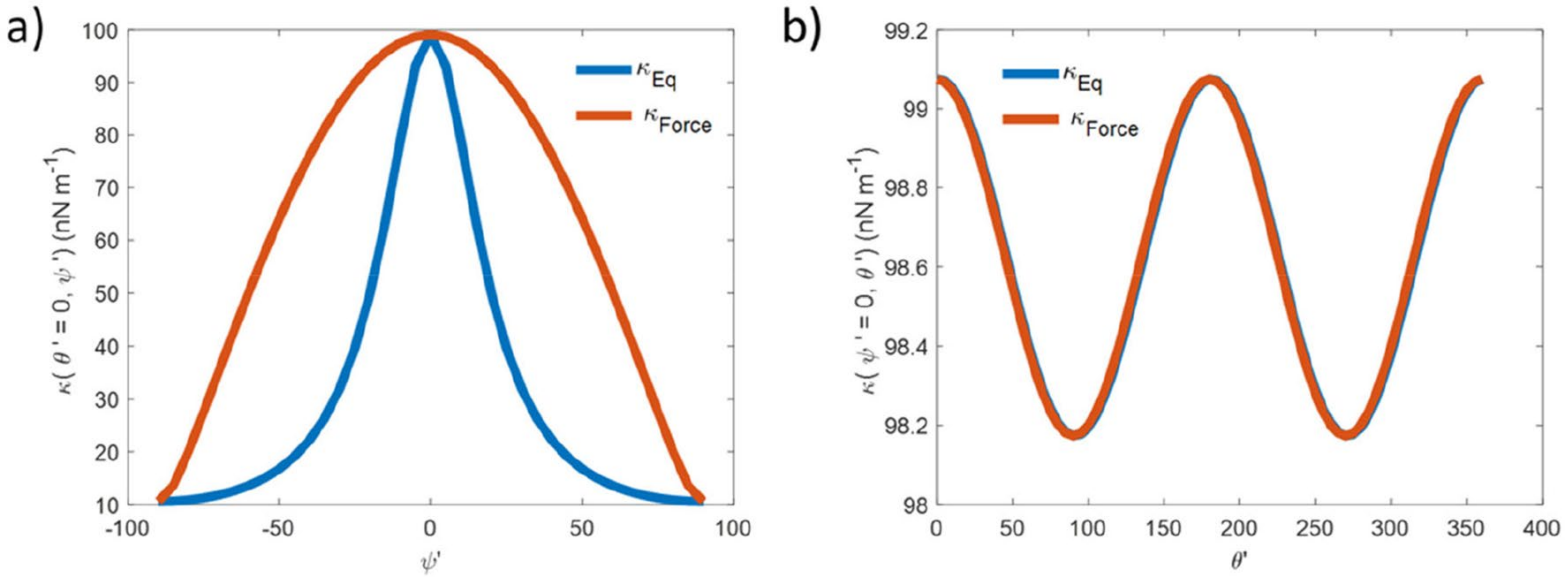
*κ* calculated using Eq. 2 (blue) and Eq. 4 (red), plotted as a function of a) *ψ*′ and b) *θ*′

The values of the trap strength obtained by using Eq. 2 (${\kappa }_{Eq}\left({\theta'},{\psi'}\right)$) and Eq. 4${(\kappa }_{{\text{Force}}} \left({\theta'},{\psi'}\right))$ are shown in Fig. 2a. Except for at ${\psi'}=0^\circ , 90^\circ$, ${\kappa }_{{\text{Force}}} \left({\theta'},{\psi'}\right)$ exceeds ${\kappa }_{Eq}\left({\theta'},{\psi'}\right)$ due to the anisotropy. For completeness, Fig. 2b shows the same parameters but plotted as a function of *θ*’, where we see very little variation in either value, and almost perfect agreement between them due to the lack of anisotropy in the *x*′-*y*′ plane (see Fig. 1e).

In common with all MOT measurements, this technique relies upon acquiring enough data points over a long time, so that the bead explores the entire volume of the optical trap (Smith et al. 2023). Additionally, this method is appropriate only for the simple case of single beam optical traps, with only one point of focus, as used for the majority of hybrid MOT measurements.

### Measuring the viscosity of fluids

For a bead of radius *a* suspended in a Newtonian fluid of viscosity $\eta$ and subjected to a trapping force (proportional to $-\kappa r$), the normalised position autocorrelation function (NPAF) can be used to calculate the fluid viscosity (Tassieri, et al. 2015):

5 $$NPAF\left(\tau ,{\theta'},{\psi'}\right)=\frac{\langle r\left({\tau }_{0},{\theta'},{\psi'}\right) r({\tau }_{0}+\tau ,{\theta'}, {\psi'})\rangle }{\langle {r}^{2}({\theta'}, {\psi'})\rangle }={\text{exp}}\left(-\frac{{\kappa }_{Eq}\left({\theta'},{\psi'}\right)\tau }{6\pi \eta \left({\theta'},{\psi'}\right)a}\right)$$

where $\tau$ is the lag-time. The NPAF is closely related to the particle normalised mean squared displacement (NMSD):

6 $$NPAF\left(\tau ,{\theta'},{\psi'}\right)=1-NMSD\left(\tau ,{\theta'},{\psi'}\right)=1-\frac{ <MSD\left(\tau ,{\theta'},{\psi'}\right)>}{2<{r}^{2}\left({\theta'},{\psi'}\right)>}$$

where $MSD\left(\tau ,{\theta'},{\psi'}\right)=<{r}^{2}\left(\tau ,{\theta'},{\psi'}\right)>$. By combining Eqs. 2, 5, and 6, one obtains:

7 $$MSD\left(\tau ,{\theta'},{\psi'}\right) = \frac{2{k}_{B}T}{{\kappa }_{Eq}({\theta'},{\psi'})} \left(1- {\text{exp}}\left(-\frac{{\kappa }_{Eq}\left({\theta'},{\psi'}\right)\tau }{6\pi \eta \left({\theta'},{\psi'}\right)a}\right)\right)$$

It has been shown that although Eq. 7 works well when analysing trajectories from isotropic 2D data(Tassieri, et al. 2015), it may return erroneously high values of *η* if there is a high degree of anisotropy in the trajectory, as is common in 3D data (Matheson et al. 2021a). This is because (except for when $r\left(t,{\theta'},{\psi'}\right)= {z'}$ or is confined to the *x*′-*y*′ plane), the particle motion along $r(t,{\theta'},{\psi'})$ becomes correlated to motion along the directions orthogonal to $r(t,{\theta'},{\psi'})$ and Eqs. 2 and 7 become invalid. This results in the aforementioned under-estimation of ${\kappa }_{Eq}$ (see Fig. 2), and an MSD curve which is no-longer purely mono-exponential. When analysing trajectories in the $x-y$ image plane, this will not affect results provided the trap beam is aligned perpendicularly with the imaging system and *z *= *z*′. However, any misalignment of the trap beam is likely to result in this phenomenon affecting the analysis of traditional 2D MOT data. Regardless, even with perfect alignment, this artefact prohibits accurate extraction of microrheological properties for motion along the full array of available solid angles theoretically available from 3D MOT measurements.

A possible solution to this problem could be achieved by looking at very short timescales so that the bead motion is affected only by the local trapping force and not the overall trap geometry. This may be done by analysing only the MSD data at lag-times $\tau \ll 1/{f}_{C}$ and using Fick’s law for unconstrained Brownian motion:

8 $$MSD\left(\tau ,{\theta'},{\psi'}\right)=2dD\tau$$

where *d* is the dimensionality of the trajectory being analysed (*d* = 1 in this case) and *D* is the diffusion coefficient defined by the Stokes–Einstein relation:

9 $$D=\frac{{k}_{B}T}{6\pi \eta \left({\theta'},{\psi'}\right)a}$$

In the case of MOT measurements, the bead is constrained by the optical trap and the MSD of its trajectory will deviate from Eq. 8. However, for very short lag-times, Eq. 8 provides a very good estimation of the MSD of the trapped particle. This is because (to a first approximation) the confining potential of the optical trap is quadratic (Ashkin, et al. 1986). Therefore, small displacements result in only very weak restoring forces, and at short timescales, the resulting MSD will be equivalent to that of an un-trapped bead. It follows that by combining Eqs. 8 and 9 one obtains:

10 $$MSD\left({\tau }_{1},{\tau }_{2},{\theta'}, {\psi'}\right)=\frac{{k}_{B}T ({\tau }_{2}-{\tau }_{1})}{3\pi \eta \left({\theta'}, {\psi'}\right)a}$$

Therefore, by determining the gradient of the MSD for the first two lag-times (i.e., ${\tau }_{2}$ and ${\tau }_{1}$, the shortest time delays $MSD\left(\tau ,{\theta'},{\psi'}\right)$ is calculated for), one should derive a good estimation of $\eta ({\theta'},{\psi'})$, as recently corroborated by this group while measuring the apparent viscosity experienced by a bead approaching a hard interface in 3D (Mendonca, et al. 2023). However, it must be noted that, in practice, the MSD values for the earliest lag times may be erroneously high if the real displacement is less than the resolution of the tracking system. Therefore, for experimental data, we ignore the lag times for which MSD < 8 × 10^−16^ m^−2^, which is derived from the resolution of the tracking system in the *z*-direction.

However, this approach returns only a value of $\eta ({\theta'},{\psi'})$, and does not allow the frequency domain to be analysed. To gain access to the frequency domain but avoid anisotropy induced artefacts, one must identify the time range where $MSD\left(\tau ,{\theta'},{\psi'}\right)$ values are overwhelmingly governed by the trapping force acting along the direction $\widehat{r}({\theta'},{\psi'})$, and not by those acting in the direction perpendicular to $\widehat{r}({\theta'},{\psi'})$. This timescale, $\tau <{\tau }_{{\text{Threshold}}}$, will be closely related to the inverse of the ${f}_{c}$ value for the trap acting along $\widehat{r}({\theta'},{\psi'})$, ${\tau }_{c}=\frac{1}{{f}_{c}}=\frac{6\pi \eta a}{\kappa ({\theta'}, {\psi'})}$. It is possible to gain a good estimation of ${f}_{C}$ from the MSD value using ${\kappa }_{{\text{Force}}}\left({\theta'},{\psi'}\right)$, as explained hereafter.

If the trap strength were not anisotropic, the MSD value should tend towards an asymptotic value $MS{D}_{Iso}\left({\tau \to \infty ,\theta'},{\psi'}\right)= \frac{2{k}_{B}T}{{\kappa }_{{\text{Force}} }({\theta'},{\psi'})}$ and one would *not* expect to observe $MSD\left(\tau ,{\theta'},{\psi'}\right)>MS{D}_{Iso}\left({\tau \to \infty ,\theta'},{\psi'}\right)$. Therefore, if one observes that $\frac{MSD\left(\tau ,{\theta'},{\psi'}\right)}{MS{D}_{Iso}\left({\tau \to \infty ,\theta'},{\psi'}\right)}>1$ beyond a specific value of $\tau$, this strongly indicates that particle motion on this timescale is *not* consistent with an idealised system where motion is uncorrelated from forces acting in the orthogonal directions. Conversely, if one chooses the timescale where $\frac{MSD\left(\tau ,{\theta'},{\psi'}\right)}{MS{D}_{Iso}\left({\theta'},{\psi'}\right)}<1$, there can be a greater degree of confidence that the bead trajectory along $\widehat{r}({\theta'},{\psi'})$ is not being influenced by forces acting orthogonally to $\widehat{r}({\theta'},{\psi'})$.

In real experiments, the trajectory will likely begin to exhibit motion due to orthogonal forces before it reaches the $MS{D}_{Iso}\left({\theta'},{\psi'}\right)$ value, and therefore, it is worth choosing a cut-off value close to the theoretically expected plateau for an isotropic trap, but without exceeding it. In this regard, a convenient value to choose may be $0.95 MS{D}_{Iso}({\theta'},{\psi'})$, because of the following reasons: –

- It fulfils the requirement of being close to $MS{D}_{Iso}({\theta'},{\psi'})$ without exceeding it;
- Even when analysing isotropic MOT data, the data are often disregarded for rheological purposes at a point close to 95% the plateau MSD value as this tends to be close to the noise floor of the corresponding NPAF plot;
- For an isotropic trap, $0.95 MS{D}_{Iso}\left({\theta'},{\psi'}\right) \sim \left(1-{\text{exp}}\left(-\frac{\kappa \left(\theta',\psi'\right)3{\tau }_{c}}{6\pi \eta a}\right)\right)MS{D}_{Iso}\left({\theta'},{\psi'}\right)$; therefore, by using this value as a cut-off, one may relate this time-range to $3{\tau }_{c}=1/{3f}_{c}$.

To elaborate on point c, one would expect the mean squared displacement value to be at 95% of $MS{D}_{Iso}\left({\theta'},{\psi'}\right)$ at $\tau = {3\tau }_{c}$ *if* the trajectory is mainly influenced by the trap force acting along $\widehat{r}({\theta'},{\psi'})$; i.e.:

11 $$MSD\left({\tau }_{{\text{Threshold}} }= 3{\tau }_{c},{\theta'},{\psi'}\right)=MS{D}_{Iso}\left( {\theta'},{\psi'}\right)\left(1-{e}^{-3}\right)= 0.95\frac{2{k}_{B}T}{{\kappa }_{{\text{Force}}}\left({\theta'},{\psi'}\right)}$$

Therefore, the point at which Eq. 11 is satisfied seems a sensible first approximation for the maximum time range to analyse the MSD values, i.e., $MSD\left({\tau }_{{\text{Threshold}}},{\theta'},{\psi'}\right)=0.95 MS{D}_{Iso}\left({\theta'},{\psi'}\right)$. By combining all the above equations, one obtains the following:

12 $$MSD\left(\tau ,{\theta'},{\psi'}\right)=\frac{2{k}_{B}T}{{\kappa }_{{\text{Force}}}\left({\theta'},{\psi'}\right)}\left(1-{\text{exp}}\left(-\frac{{\kappa }_{Force}\left({\theta'},{\psi'}\right)\tau }{6\pi \eta \left({\theta'},{\psi'}\right)a}\right)\right), \forall \tau <{\tau }_{{\text{Threshold}}}$$

**Fig. 3:**
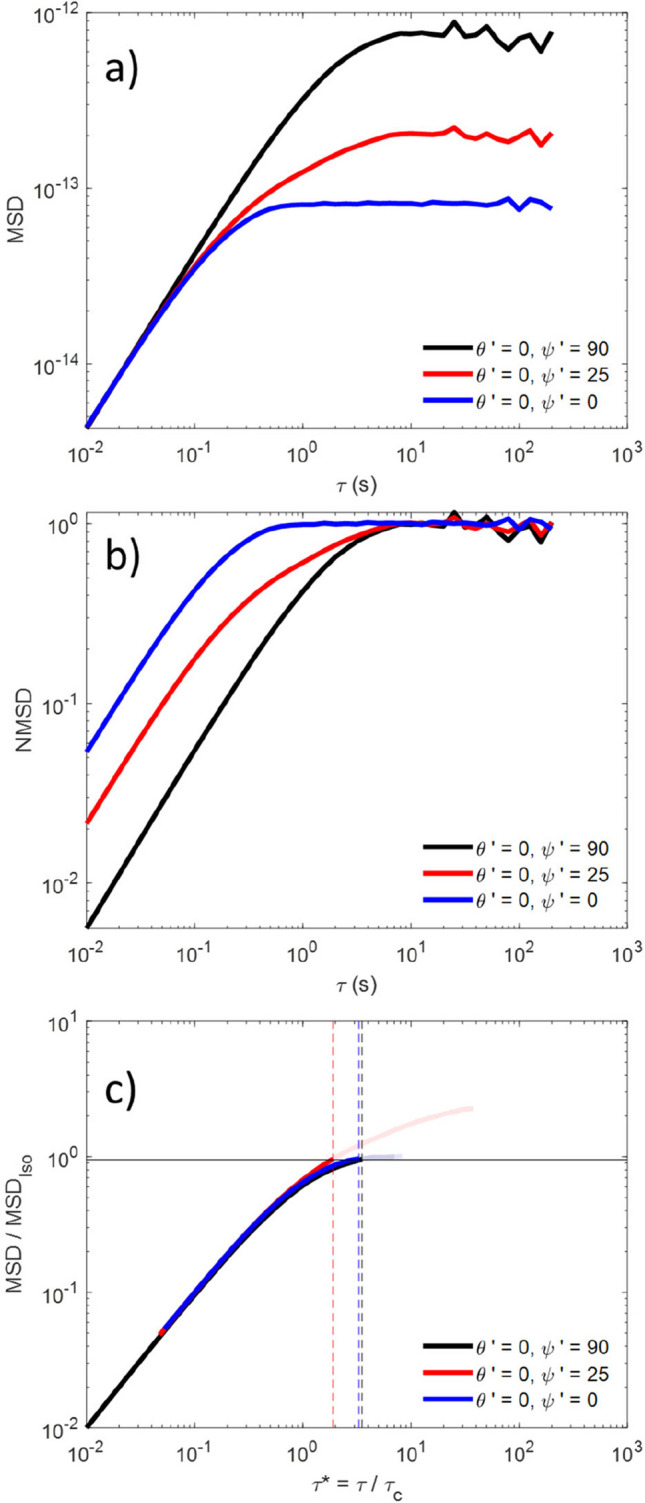
**a** MSD and **b** NMSD data versus delay time for trajectories aligned along *x*′ (blue), *z*′ (black), and at an angle 25° from *x*′ (red). **c** $MSD/MS{D}_{Iso}$ versus $\tau /{\tau }_{c}$ for the same trajectories, with the solid horizontal line marking 0.95 and the dashed vertical lines representing the ${\tau }_{Threshold}$ point for each data set. Bold lines are used for data < ${\tau }_{c}$, with fainter lines used for data after this point

Figure 3 a shows the MSD plots for motion along three different directions for the simulated data reported in Fig. 1. These correspond to ${z'}= r\left({\theta'}=0, {\psi'}=90\right)$ (black), ${x'}= r\left({\theta'}=0, {\psi'}=0\right)$ (blue), and an intermediate direction $r\left({\theta'}=0, {\psi'}=25\right)$ (red). When data are normalised by twice their variance ($2<{r}^{2}\left({\theta'},{\psi'}\right)>$), Fig. 3b is generated. From these NMSD plots, it is apparent that the red curve does not have a mono-exponential form, unlike the black and blue ones. However, this is not in itself sufficient to confirm the effects of trap anisotropy. A highly non-mono-exponential MSD curve with more than one plateau may be produced by a viscoelastic material in a symmetric trap (Tassieri, et al. 2010), which may be hard to distinguish from the effect of trap anisotropy (Matheson et al. 2021a). Importantly, the approach developed in this paper is based on the absolute value of the MSD curve rather than its shape, thus avoiding confusion between the side effect of trap anisotropy and the genuine viscoelastic response of the materials.

In Fig. 3c, the same data as in Fig. 3a are plotted, but normalised not to $2<{r}^{2}\left({\theta'},{\psi'}\right)>$, but to the $MS{D}_{Iso}({\theta'},{\psi'})$ value. Moreover, the time axis has been made the dimensionless value ${\tau }^{*}= \frac{\tau }{{\tau }_{C}}$, where ${\tau }_{C}$ has been calculated using the $\eta$ value employed as simulation input. As a result, the early time data collapses onto a master curve, as expected for an isotropic fluid (Matheson, et al. 2021b; Tassieri, et al. 2015). The solid horizontal line in Fig. 3c marks 0.95 $MS{D}_{Iso}({\theta'},{\psi'})$, i.e., the point at which we set ${\tau }_{{\text{Threshold}}}$ for each data set, whereas the dashed vertical lines represent the ${\tau }_{{\text{Threshold}}}$ points, colour-coded to match each trajectory. In Fig. 3c, the curves for $\tau > {\tau }_{Threshold}$ have been plotted more faintly than those for $\tau < {\tau }_{{\text{Threshold}}}$; as it is known that for $\tau > {\tau }_{{\text{Threshold}}}$, data may induce artefacts to further analysis. Notably, for the data aligned along the semi-axis of the trap (black and blue lines), ${\tau }_{{\text{Threshold}}}$ is very close to the $3{\tau }_{c}$ value we would expect for a Newtonian fluid, which is a good indication of the robustness of this technique. For the data not aligned with the ${x'}-{y'}$ plane or $z'$ (red line), however, ${\tau }_{{\text{Threshold}}}$ occurs earlier which is an indication that the trajectory along this direction has become correlated with the bead motion in an orthogonal direction.

### Frequency domain analysis

In order to measure the frequency-dependent viscoelastic properties of materials, the methodology outlined by Smith et al. (2021) provides a valuable starting point. Briefly, the Fourier transform of the normalised mean squared displacement ($\widehat{{\text{NMSD}}}$) is used to calculate the complex shear modulus of the material, ${G}^{*}\left(\omega ,{\theta'},\psi'\right)$:

13 $${G}^{*}\left(\omega ,{\theta'},\psi'\right)\frac{6\pi a }{\kappa \left({\theta'},{\psi'}\right) }=\frac{1}{i \omega \widehat{{\text{NMSD}}}(\omega ,{\theta'},{\psi'})}$$

The Fourier transform to calculate $\widehat{{\text{NMSD}}}(\omega ,{\theta'},{\psi'})$ is performed up to the point where ${\text{NMSD}}(\tau ,{\theta'},{\psi'})$ approaches its plateau value, where it will comply with the boundary condition $\frac{dMSD\left(\tau \to \infty ,{\theta'},{\psi'}\right)}{d\tau }=0$. Typically, this means performing a Fourier transform on the data up to the range of $0.95<NMSD\left(\tau ,{\theta'},{\psi'}\right) <1$. As outlined previously, extending the time range over which the transform is applied to longer times may generate artefacts in the output, while curtailing the data too early may introduce other artefacts due to the function not being close enough to its boundary conditions.

By replacing NMSD with MSD in Eq. 13 and by using Eq. 2 to remove the dependency on $\kappa$, one obtains the following relationship:

14 $${G}^{*}\left(\omega ,{\theta'},\psi'\right)=\left(\frac{{k}_{B}T }{3\pi a }\right)\frac{1}{i \omega \widehat{{\text{MSD}}}(\omega ,{\theta'},{\psi'})}=G'\left(\omega ,{\theta'},{\psi'}\right)+iG''\left(\omega ,{\theta'},\psi'\right)$$

where the real part and the imaginary parts are the storage and loss moduli of the material, respectively, and are a measure of the elastic and viscous character of the material.

In order to find the frequency domain where the moduli will be free from artefacts induced by trap anisotropy (via $MSD\left(\tau ,{\theta'},{\psi'}\right)$), the same basic approach used for Eq. 11 may be adopted. Indeed, by finding the value of ${\tau }_{{\text{Threshold}}}$ where $MSD\left({\tau }_{{\text{Threshold}}},{\theta'},{\psi'}\right)=0.95 MS{D}_{Iso}\left(\theta',{\psi'}\right)$, and taking its inverse, one may identify a cut-off frequency ${f}_{{\text{Threshold}}}=\frac{1}{{\tau }_{{\text{Threshold}}}}$, which can be used as a low-frequency cut-off value. In the case when the bead trajectory along the direction $\widehat{r}({\theta'},{\psi'})$ is independent of the forces acting in the orthogonal direction, then one would obtain: ${f}_{{\text{Threshold}}}\sim \frac{1}{{3\tau }_{c}}=\frac{{f}_{c}}{3}$.

Finally, for a Newtonian fluid, the complex viscosity ${\eta }^{*}\left(\omega ,{\theta'},\psi'\right)$ can be obtained using the following equation:

15 $$\left|{\eta }^{*}(\omega ,{\theta'},\psi')\right|\equiv \frac{\left|{G''}\left(\omega ,{\theta'},{\psi'}\right)\right|}{\omega }$$

By determining the frequency at which $f>{f}_{{\text{Threshold}}}$, the region where Eq. 15 holds can be identified and the fluid viscosity determined.

## Results and discussion

### Simulated data

To validate the analytical framework introduced above, simulated trajectories were used to avoid un-accounted sources of anisotropy in the *η* value. The simulated trajectory of a bead in water (i.e., a homogenous Newtonian fluid) shown in Fig. 1b and discussed in the previous section will be used for this purpose.

**Fig. 4:**
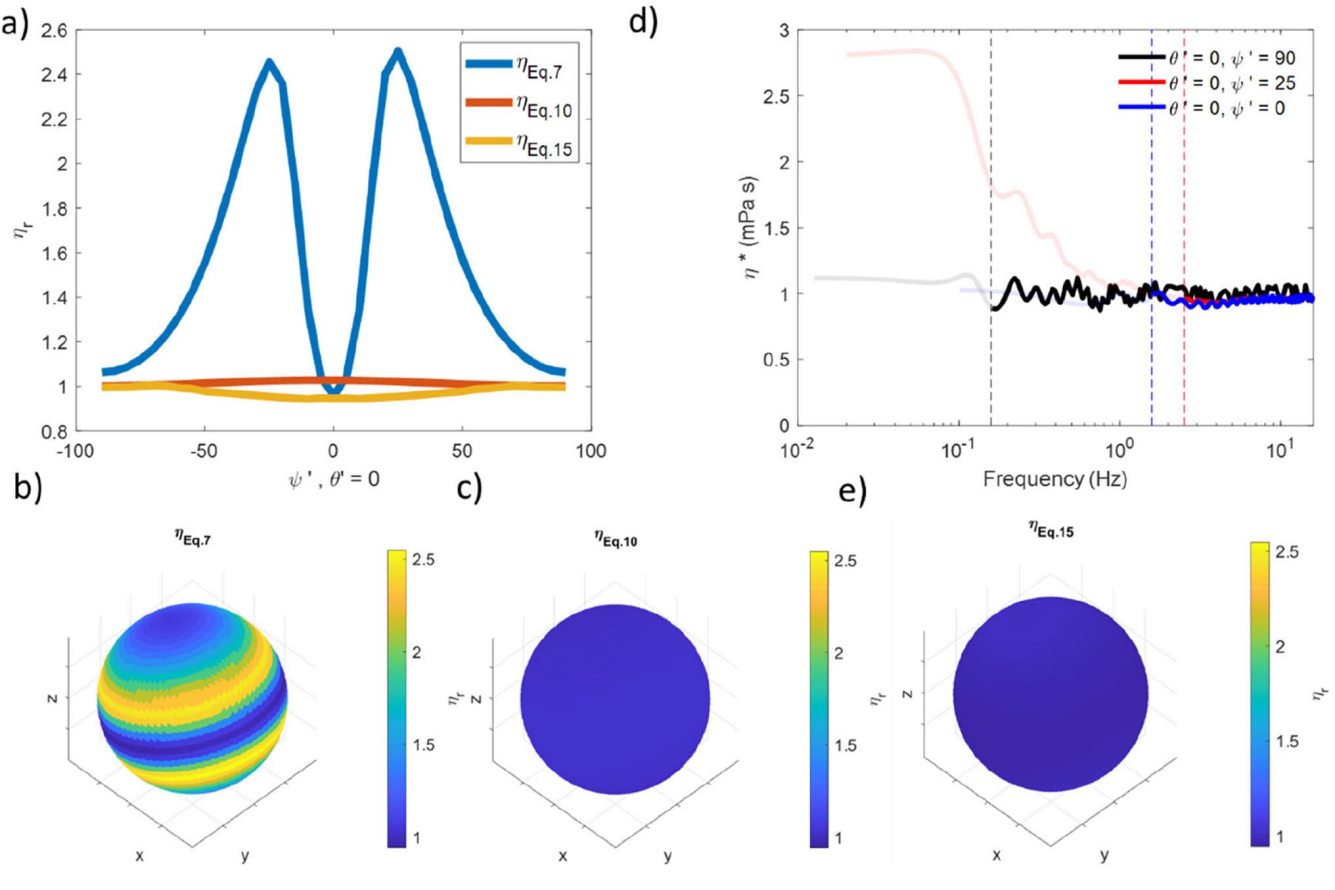
**a** Relative viscosity as a function of *ψ*′ in the *x*′-*z*′ plane calculated via Eq. 7 (blue), Eq. 10 (red), and Eq. 15 (gold). **b** A spherical surface shaded to represent viscosity for motion along the vector running from the origin to the shaded point, calculated using Eq. 7, **c** as **b** but calculated using Eq. 10, **d** η* with data for *f* < *f*_Threhsold_ shown as a faint line and *f* > *f*_Threhsold_ shown as bold for $\theta' = 0^\circ , \psi' = 0^\circ , 25^\circ , 90^\circ$ (blue, red, black, respectively). ${f}_{Threshold}$ is represented as a vertical dashed line for each data set. **e** as **b** and **c** but calculated using the mean of η* obtained from Eq. 15

In order to measure fluid viscosity with MOT measurements, typically Eqs. 5–7 are employed. However, as demonstrated previously, this may result in erroneous results due to trap shape anisotropy. In Fig. 4a are the relative viscosity values (drawn as a blue line in the plot) obtained via Eq. 7 by holding ${\theta'} = 0$ and varying ${\psi'}$ (i.e., by resampling the data in the *x*′–*z*′ plane). Figure 4 b shows a spherical surface, where the position on the surface identifies the direction of motion being probed and the shading corresponds to the measured viscosity, as indicate by the colour bar. From Fig. 4b, it is clear that Eq. 7 returns a significant overestimation of the fluid’s viscosity, with a maximum relative value of *η*_*r*_ ~ 2.5, representing an error of ~ 150%.

Figure 4 a and c show the viscosity values obtained by means of Eq. 10. Over all angles, the average viscosity is 1.01 Pa $\cdot$ s, with a maximum error of just 3%. Hence, the accuracy in calculating the viscosity of Newtonian fluids has been significantly improved. However, this approach does not allow access to the frequency domain.

Figure 4 d, however, shows the complex viscosity vs. frequency obtained from the full frequency resolved analysis of the same data. In this graph, data below ${f}_{{\text{Threhsold}}}$ (as identified in Fig. 3) are shown as faint lines and will be unreliable because of the trap anisotropy (for data not aligned along *z*′ or *x*′–*y*′ ) and noise in the plateau region of the NMSD plot (all data), whereas data at higher frequencies than this threshold should return valuable results.

To obtain a single value of the Newtonian viscosity, one may take the mean of the data for $f>{f}_{{\text{Threshold}}}$. These values are shown in Fig. 4a (yellow line) as a function of angle in the $x-z$ plane and in 3D are shown in Fig. 4e. Notably, over the range of explored angles, this approach returns a mean value of ${\eta }_{r}= 0.98$, with a maximum mean squared error of only 5.7%. It is thus apparent that by using ${\kappa }_{{\text{Force}}}$ and the curtailed MSD data the accuracy of the calculated viscosity over the full angular range has been significantly improved compared to Eq. 7, while still allowing *η* to be probed over a broad frequency range unavailable using Eq. 10.

### Experimental data

**Fig. 5:**
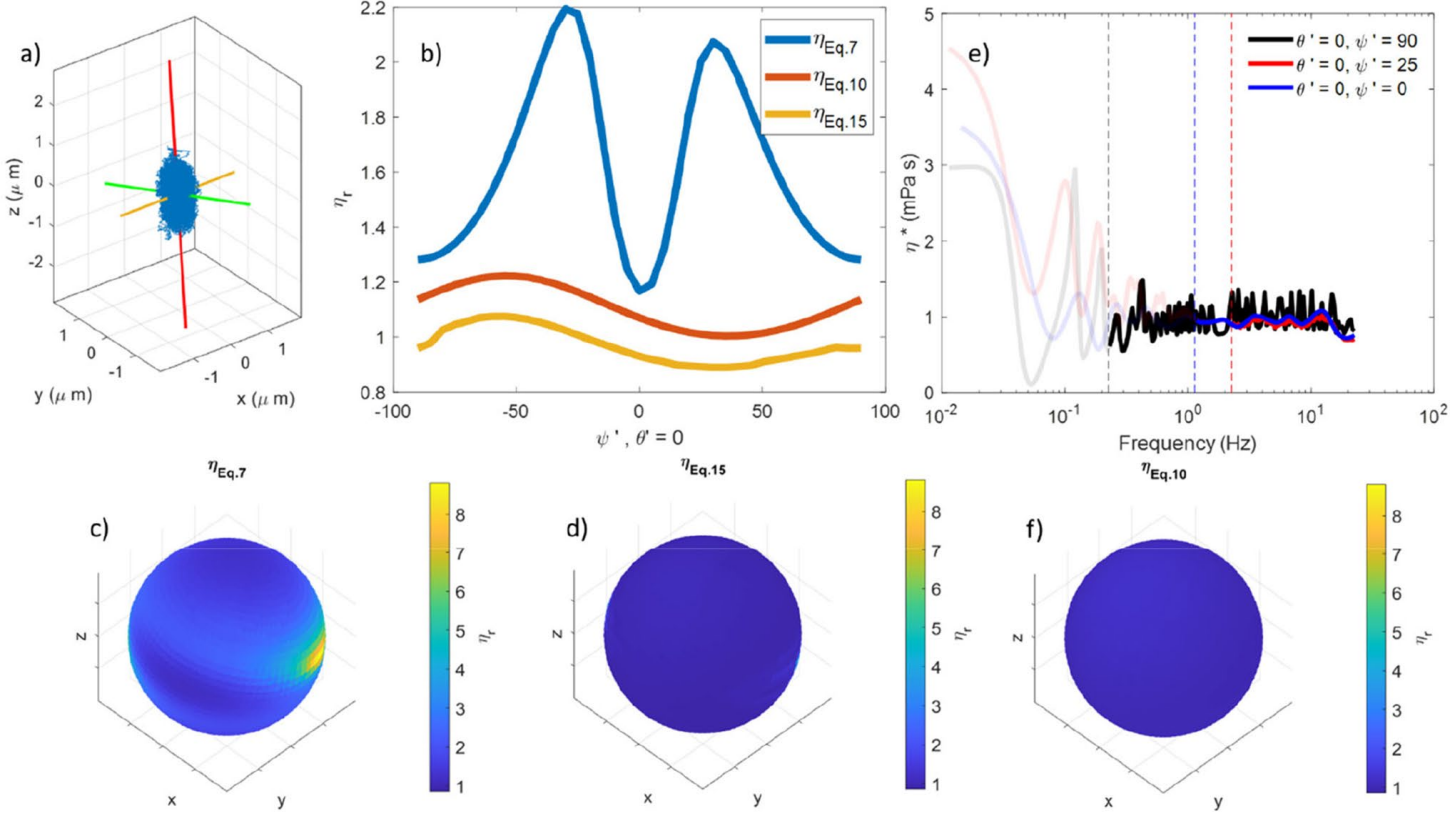
**a** The scatter plot for an optically trapped bead in water, the *x*′, *y*′, and *z*′ axes are shown in green, yellow, and red, respectively. **b** Relative viscosity as a function of *ψ*′ in the *x*′-*z*′ plane calculated via Eq. 7 (blue), Eq. 10 (red), and Eq. 15 (gold). **c** A spherical surface shaded to represent viscosity for motion along the vector running from the origin to the shaded point, calculated using Eq. 7, **d** as **b** but calculated using Eq. 10. **e** Viscosity vs *f* obtained via Eq. 15 for *θ*′ = 0°, *ψ'* = 0°, 25°, 90° (blue, red, black, respectively). Data for *f* < *f*_threshold_ shown as a faint line and *f* > *f*_threshold_ shown as bold. **f** as **c** but calculated using Eq. 15

Having demonstrated the effectiveness of this method on simulated data, attention is turned to experimentally obtained data. Figure 5 a shows the trajectory of an optically trapped bead in water, with *z* and *z*′ clearly not aligned.

In Fig. 5b, we report the viscosity calculated using each of the three methodologies introduced earlier as the data is sampled through the *x*′-*z*′ plane. As for the simulated data, Eq. 7 gives a highly anisotropic and erroneous viscosity output with Eqs. 10 and 15 showing much less viscosity variation over the range of explored angles. Indeed, over a rotation of *ψ'* in the *θ'* = 0 plane, by using Eq. 7, we see a maximum error in this plane of ~ 120%, whereas for Eq. 10, the error is ~ 22%, while for Eq. 15, it is just 11%. In Fig. 5c, the complex viscosity vs. frequency is plotted for *ψ'* = 0°, 25°, and 90°. Once again, the threshold frequencies (represented by the vertical dashed lines) seem to match well the point at which the viscosity begins to deviate from its otherwise very flat and constant value of ~ 1 mPa $\cdot$ s. Thus, corroborating the effectiveness of the analysis method introduced in this work.

In real experiments, it is very easy to unknowingly introduce tilt into the propagation of the trap beam. Indeed, it was not deliberately added to the data shown in Fig. 5 (which showed a fairly isotropic cross section in the *x*–*y* plane), the tilt was only apparent when the data was analysed in 3D. Interestingly, this suggests that without fully measuring and analysing MOT data in 3D, experimentalists may be unaware of slight misalignments of the *z* and *z*' axes (which will result in an overestimation of viscosity in the *x*–*y* plane), giving a further demonstration of the value of a full 3D analysis. Previously, we demonstrated that detailed resampling of 2D projections of the scatter plot to find *z*′ may help to correct for this (Matheson et al. 2021a), but the principal component analysis approach outlined in this work samples the entire 3D space simultaneously, making this faster and more precise. However, even if *z* and *z*′ are perfectly coincident, the trap anisotropy would prevent accurate measurement of viscoelastic properties in directions not aligned parallel or perpendicular with *z*'.

**Fig. 6:**
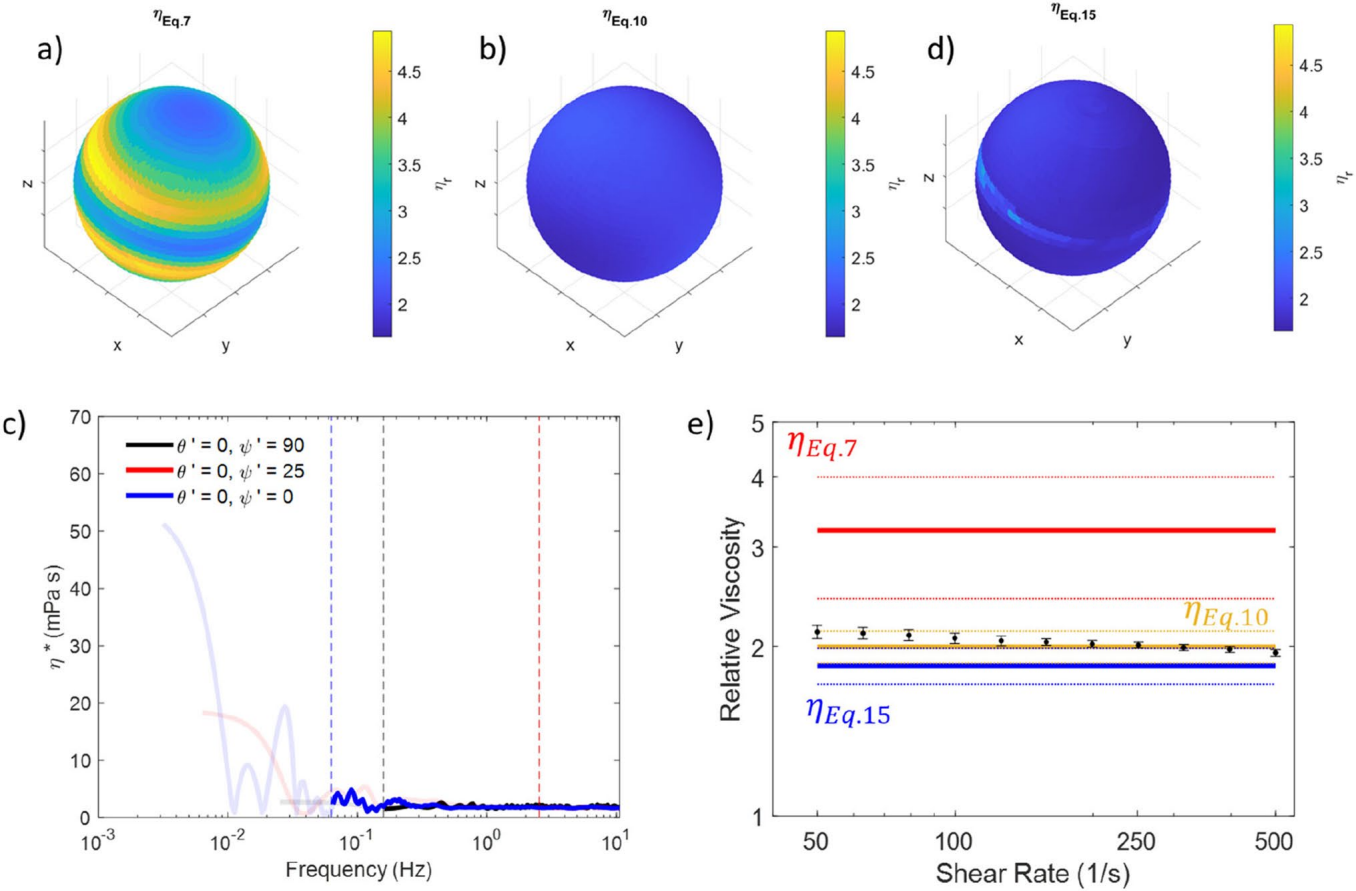
Results for a 0.5% gelatine solution. **a** A spherical surface shaded to represent viscosity for motion along the vector running from the origin to the shaded point, calculated using Eq. 7, **b** as **a** but calculated using Eq. 10. **c** Viscosity vs *f* obtained via Eq. 15 for *θ*′ = 0°, *ψ*′ = 0°, 25°, 90° (blue, red, black, respectively). Data for *f* < *f*_threshold_ shown as a faint line and *f* > *f*_threshold_ shown as bold. **d** as **a** and **b** but calculated using Eq. 15. **e** Bulk rheology results for a 0.5% gelatine solution (black symbols). Mean viscosity values obtained using Eq. 7 (red), Eq. 10 (gold), and Eq. 15 (blue) are shown as solid lines for comparison, with dotted lines to show the standard deviation around this mean

To demonstrate the efficacy of the proposed framework on a sample with higher viscosity, Fig. 6 shows experimental data obtained for a 0.5% gelatine in water solution. Figure 6 a shows the viscosity of the solution obtained by using Eq. 7, returning a relative viscosity ranging from 2.3 to 4.9. Whereas, we obtain a relative viscosity ranging from 1.7 to 2.3 by means of Eq. 10. Figure 6 c shows the ${\eta }^{*}$ vs frequency plot for this sample at a range of angles. Again, we see that at lower frequencies below ${f}_{{\text{Threshold}}}$ significant deviation between the viscosity obtained at differing angles, but close agreement at higher frequencies. This is borne out in the relative mean viscosities shown in Fig. 6d which vary between 1.7 and 2.9. To allow for a ground truth, bulk rheology values were obtained for a 0.5% gelatine in water solution, as shown in Fig. 6e. Averaging over shear rate for all seven measurements, this returned a mean relative viscosity value of 2.0 ± 0.1, which when compared to the value of 3.2 ± 0.8 for the mean over all space obtained with Eq. 7, and to the value of 2.0 ± 0.1 obtained via Eq. 10, and 1.8 ± 0.1 via Eq. 15, it confirms the improved accuracy achieved by the latter two approaches.

**Fig. 7:**
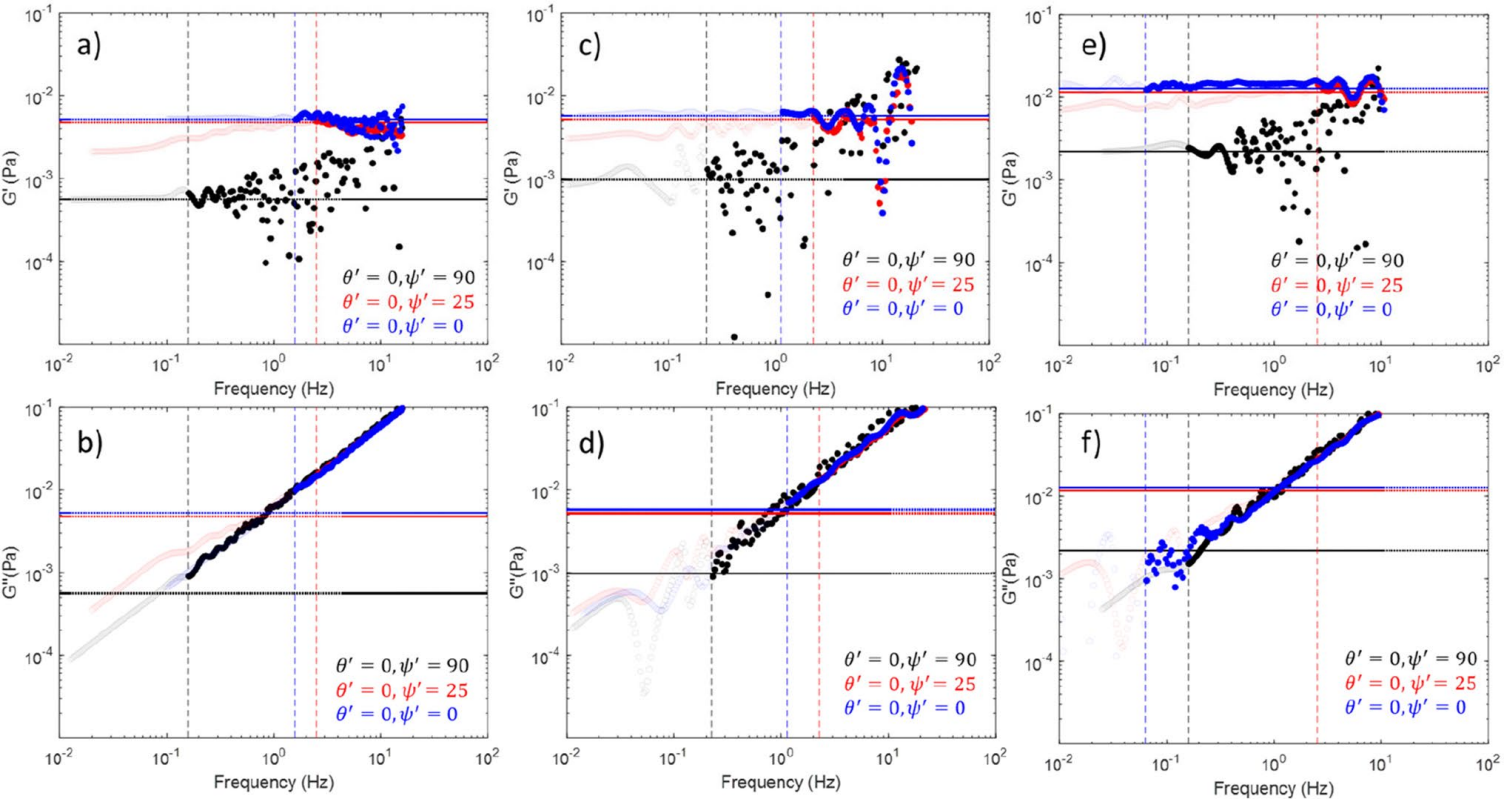
*G*′ (panels **a**, **c**, and **e**) and *G*″ (panels **b**, **d**, and **f**) as a function of frequency. Data for the simulated bead (shown in Figs. 1–4) is in panels **a** and **b**. Data for the bead trapped in water (shown in Fig. 5) are in panels **c** and **d**. Data for the bead in a 0.5% gelatine solution (shown in Fig. 6) is included in panels **e** and **f**. Dashed vertical lines correspond to ${f}_{threshold}$ values for each dataset, dotted horizontal lines are equal to $G'\left({\theta'},{\psi'}\right)={\kappa }_{Force}({\theta'},{\psi'})/(6\pi a)$. Data points at frequencies above threshold are shown in bold; data points below threshold are shown faintly. The colours black, red, and blue are used to correspond to the data at ${\theta'}=0, {\psi'}=90$, ${\theta'}=0, {\psi'}=25$, and ${\theta'}=0, {\psi'}=0$, respectively. The dashed and dotted lines are colour-coded to match the symbols of the data set they refer to

We now wish to explore the effectiveness of the proposed framework in terms of the viscoelastic moduli. Figure 7 a and b show the moduli values for the simulated data shown in Figs. 1–4, Fig. 7 c and d show the moduli for the bead trapped in water shown in Fig. 5, and Fig. 7 e and f show the moduli for the bead in a 0.5% gelatine solution shown in Fig. 6. We see that far all the data presented, in the region above ${f}_{{\text{Threshold}}}$ $G''$ (bold circles, lower row of panels) is independent of angle, as expected for an isotropic fluid. The values of $G''$ below this frequency (faint circles, lower row of panels) are much noisier and show significant variation with angle. The results for $G'$ (upper row of panels) match well to ${\kappa }_{{\text{Theory}}}/(6\pi a)$ (marked as a horizontal dotted line) as expected for an optically trapped bead in a viscous fluid (Tassieri, et al. 2010). It should be noted that for the data aligned along *z*′ in particular, the $G'$ values are very noisy, as the $G'$ values here are close to the resolution of what can be achieved with the tracking system. Nevertheless, this is a further demonstration of both the imaging system and the tracking and analysis methodology, as it is able to return results which match what is theoretically expected. A next step for this analytical method will be to turn attention towards samples which have a higher value of $G'$ and a more pronounced viscoelastic character.

## Conclusions

Although there are many potential pitfalls when analysing anisotropic microrheology data obtained in 3D microrheology with optical tweezers, this work demonstrates an analytical solution that ensures accurate characterisation. The key to this is the identification of the time regimes where the MSD along a given direction shows minimal correlation with the strength of the trap acting along directions orthogonal to this. This approach is corroborated by analysing both simulated and experimental data. When analysing data using the traditional means of MOT analysis, the level of trap anisotropy typical in a 3D trap is shown to over-estimate the fluids’ viscosity by a factor of 2.5 along certain directions. By using the methods detailed, here, it is possible to reduce this error down to < 6%, either by fitting to only the very early times of the particles’ mean square displacement to Fick’s law for Newtonian fluids, or by analysing the fluids’ frequency dependent response over a broader time-scale. The analytical framework presented offers the opportunity to significantly enhance 3D MOT measurements, allowing for full angularly resolved 3D viscoelasticity mapping of complex media, and the exploration of the materials’ frequency response in any arbitrary solid angle. For a homogenous material, this has the added benefit of increasing the frequency range over which the microrheology characterisation can be made by taking advantage of the different trap strengths in x and y compared to z.

## Data Availability

The datasets used in this study are available from the corresponding author on reasonable request.
